# Periscope of Dermatology

**Published:** 1899-04-29

**Authors:** 


### Periscope of Dermatology.

**Eczema.**—Leslie Roberts,[1] in an exhaustive paper on the nature of eczema, describes the pathology of the disease as a reaction of the mesoderm to epithelial irritation, or to some irritant seated in the epithelium, and leading thereby to certain temporary alterations in the nature of the epithelial cells; the epithelial irritation may be due to direct irritants, to the sun's rays, to chafing, to nerve influences, to toxic products of digestion, or to the products of micro-organisms in the skin. The continual irritation by any cause leads to chemical changes in the epithelium and in the lymph. He is not inclined to believe in the parasitic origin of all cases of eczema, but allows that the disease may result from the irritation of bacteria. Finally, he considers that the epithelial stimuli, leading to eczema, practically never act singly, but as complexes. Whitfield[2] considers that seborrhoic eczema starts in the follicles, and is due probably to parasitic infection, although he does not consider that this has been proved; with regard to simple eczema he does not agree at all with the idea of its parasitic origin, and further does not recognise any special form as of a gouty nature. With regard to treatment, in the parasitic varieties he commences with strong antiseptic remedies, but in simple eczema he gives soothing lotions, with just sufficient antiseptic power to prevent growth of any accidental contamination. Unna[3] states that eczema of the hands is a most stubborn disease, especially in persons who do manual work, such as bricklayers, grocers, bakers, and typesetters, all of whom wash their hands frequently while they are at work. It is not the washing, however, which has a bad effect, but the exposure of the hands to the air immediately afterwards, whereby the horny layer of the epidermis is dried and becomes the seat of little fissures that serve as avenues of entrance for dust and other injurious matters. On this account it is necessary that the hands should be washed only at night, and then covered at once with an impermeable coating of ointment. The hands should not be washed in the morning, but some firm ointment or paste should be frequently applied; for purposes of cleanliness, as before eating, simple rubbing with oil, followed by a dry wipe, suffices; oiling should never be omitted after using soap. Careful attention to these details will cure eczema of the hands even in kitchen-maids and washerwomen. Edlefsen[4] has found a successful mode of treatment of chronic eczema of the hands. This disease chiefly affects washerwomen, but not unfrequently women of the better classes. He orders a paint consisting of iodine 1 part, iodide of potassium 2·5 parts, and glycerine 20 parts; this paint is applied every evening and the hands are enveloped in lint. The irritation is always relieved, and in fourteen days the disease is generally cured, and this too when other remedies have failed. In the more obstinate cases, boracic ointment was applied in the morning, and iodine paint in the evening. Esler[5] describes the case of a man, who, on the day following the application of iodoform to a wound of the ankle, developed an acute eczematous eruption, which commenced near to the wound and spread up to the knee. The patient stated that on two other occasions the use of iodoform had occasioned a similiar eruption, and that both his father and his sister were similarly affected by iodoform. Hall[6] records the case of a chemical laboratory attendant who developed an attack of acute eczema whenever he used a drug called phenyl hydrazin. The rash resembled an ordinary recurrent eczema, and its origin was only detected by accident after some years. The last attack occurred after some of the chemical had accidentally been used in the laboratory. Buckley[7] lays great stress on the neurotic origin of eczema; he points out that the incidence of the disease is greatest between the ages of 20 and 40, which is the period at which the strain of life is greatest. The following conditions may be associated with neurotic eczema: (1) Neurasthenia; (2) nervous and mental shock; (3) reflex phenomena, cutaneous and peripheral; and (4) neuroses, structural and functional. In neurasthenia the eruption usually begins on the hands and face, being vesicular in the former position, and erythematous in the latter. Both constitutional and local treatment must be employed. Nerve tonics are indicated, and the irritation must be relieved by antipyrin or antifebrin, opium being useless. As regards diet, vegetable substances and phosphates should be given in excess. For local treatment, calamine lotion, or a brief application of very hot water, is of service. Greder[8] records a case of chronic eczema of 31 years' duration, and associated with elephantiasis of the legs, which was cured after a few days treatment with naftalan.

**Lupus, &c.**—Wild,[9] in a paper on cutaneous tuberculosis, points out that changes of a protective nature occur, in the first instance, after the tubercle bacillus has effected an entrance into the skin; these consist of increased hyperæmia and activity of the cells. He believes that most cases of cutaneous tuberculosis are caused by direct inoculation; thus the ulcer near the nose and mouth of a phthisical patient is inoculated by the tubercular sputum. Again, the verrucca necrogina, which is found on the hands of butchers and dissecting-room porters, is distinctly inoculated. Finally, in ordinary lupus, an account of inoculation can sometimes be obtained. As examples he cites the following: Women have developed lupus of the hands by nursing a tuberculous husband, or by washing his linen; a baby has developed lupus by being kissed by a phthisical nurse. Again, lupus has appeared in a tattoo mark which had been moistened by the lips of a phthisical operator, and it has also occurred after piercing of the ears. With regard to lupus erythematosus, he believes in the tubercular nature of the disease, and considers that it bears the same relation to lupus vulgaris as fibroid phthisis does to ordinary phthisis. Walker,[10] in recording a case of lichen scrofulosorum, agrees with the tubercular nature of the disease; but considers that it is caused, not by the bacilli itself, but by its toxins. He points out that these cases get well even when left alone, a result which does not occur in other tubercular processes. Adamson[11] records a case of multiple cutaneous lupus occurring after measles; the patient was a boy, aged three, who had suffered from measles one year before, and the tubercular eruption had developed during the attack. The lesions were raised purplish-brown patches, varying in size from a tenth to a quarter of an inch in diameter. A year later they had almost disappeared, but the child was suffering from a tubercular hip. Brousse and Arden-Delteil[12] describe the case of a patient, aged 16, who had suffered from polymorphous tuberculosis of the skin since the age of four. The lesions were as follows: (1) Tuberculosis verrucosus of the right foot and right leg; (2) scrofulo-tuberculous gummata of the left side of the face, involving the submaxillary regions, and the front of the left sterno-mastoid; and (3) a patch of true lupus on the left cheek. The disease had improved after treatment by curetting, igni-puncture, and tonics. Menau[13] records the case of a girl aged nine, who had suffered from various tubercular affections; since the age of three she had attacks of rose-red spots, the size of a millet seed, surmounted by a purulent crust; when this disappeared slightly depressed scars were left. The eruption affected the face and the extremities; no bacilli were found, so the author considers that the eruption was caused by the absorption of toxins of the bacillus. Finsen[14] has treated 59 cases of lupus by means of concentrated chemical light rays. Of these cases 23 were cured, and of the remainder, all but one showed improvement. The rays employed are the most refracted ones of the spectrum of the sun or of an electric arc lamp; the other rays are excluded by the passage of the light through a hollow lens containing methylene blue or sulphate of copper in solution. The rays are then focussed on the spot to be treated, and are applied to an area of from one to three square inches, for from one to two hours every day. The treatment lasts for a few days or for several weeks. The rays are rendered more powerful if the nodule is made anæmic by pressure. This is effected by applying a glass disc close to the part and keeping it fixed there by elastic bands. Hyperæmia, vesicles, and desquamation are produced; ulcerations cicatrise, and the raised parts become level. The light from incandescent lamps is useless. Bang[15] also advocates this method, and states that with an arc lamp, the rays from which are rendered parallel by two lenses of rock crystal, bacteria can be destroyed in a test-tube of rock crystal within one minute. He applies the rays in the same way as Finsen, save that compression of the lupus patch is effected by a nurse pressing on the skin with an apparatus, composed of two plates of rock crystal, between which a regulated stream of cold water flows. In this way too great heat is avoided. The author considers that the treatment is most valuable, and its only drawback is its slowness.

[1] Brit. Jour. of Derm., Jan., 1899, p. 72. [2] Lancet, Feb. 25, 1899, p. 518; Brit. Med. Jour., Feb. 25, 1899, p. 473. [3] Mon. fur prakt. Derm., No. 26, 1898, p. 547. Edin. Med. Jour., Oct., 1898, p. 379. [4] Brit. Med. Jour., April, 1899, Epit., No. 370. [5] Brit. Jour. of Derm., Feb., 1899, p. 88. [6] Brit. Jour. of Derm., Mar. 1899, p. 112. [7] Edin. Med. Jour., Oct., 1898, p. 317. [8] The Therapeutist, Dec. 15, 1898, p. 279. [9] Brist. Med. Chir. Jour., Sept., 1898, p. 241. [10] Brist. Med. Chir Jour., Sept., 1898, p. 241. [11] Brit. Jour. of Derm., Jan., 1899, p. 20. [12] Brit. Jour. of Derm., Nov., 1898, p. 421; La Presse Med., Nov., 1898, p. 189. [13] Jour. des Maladies, Cut. et Syph., April, 1898; Brit. Jour. of Derm., Nov., 1898, p. 421. [14] Brit. Jour. of Derm., Oct., 1898, p. 376. [15] Brit. Jour. of Derm., Oct., 1898, p. 377; La Presse Med., Aug., 1898, p. 65.

